# Health post readiness and its influence on mothers’ care-seeking practice for their sick children in Ethiopia

**DOI:** 10.3389/fpubh.2025.1569970

**Published:** 2025-06-16

**Authors:** Wassie Negash Mekonnen, Gizachew Tadele Tiruneh, Adugnaw Birhane, Wubegzier Mekonnen

**Affiliations:** ^1^School of Public Health, College of Health Sciences, Addis Ababa University, Addis Ababa, Ethiopia; ^2^JSI Research and Training Institute, Inc., Addis Ababa, Ethiopia

**Keywords:** health post readiness, sick children, community level care, iCCM context, care seeking

## Abstract

**Introduction:**

Infection accounts for about half of all neonatal deaths and it contributes to 37% of neonatal deaths in Sub-Saharan Africa where there is low health facility readiness and the quality of service given at health facilities is low. In this study, we assessed the influence of health posts’ readiness on the care-seeking behavior of mothers of sick young children.

**Method:**

This study analyzed data from a community-based implementation survey conducted by JSI in the two districts of Ethiopia from April 2021 to July 2022. In this study, we enrolled 4,262 and 4,081 mothers with children < 15 months at the baseline and end-line surveys, respectively, of which 508 and 359 infants were diagnosed for illness at 66 and 64 health posts at the baseline and end-line surveys. We used the Service Availability and Readiness Assessment tool to compute the facility readiness score. We used independent sample *t*-test and logistic regression to see the contributions of facility readiness for care-seeking practices of mothers. AOR at 95% CI and *p*-value < 0.05 is used to declare a statistically significant association between variables and to control the confounding.

**Results:**

In the end-line survey about 359 sick young infants were identified. And in the baseline survey, 508 young infants were ill. Most of 88.0% sick young infants sought care in the end line compared to 57.3% at the baseline (*p* < 0.001). The overall summated mean facility readiness score was 69.6%, equivalent to 49.0% of the standardized mean score. This study also highlights rich households (AOR = 2.02; 95% CI: 1.1–3.9), reaching out to health posts (HPs) equipped with materials and supplies (AOR = 1.52; 95% CI: 1.2–1.9), and ANC use (AOR = 2.35; 95%CI: 1.2–4.7) were positively associated with care seeking practice compared to their counterparts.

**Conclusion:**

The study reveals a moderate level of health post-readiness that needs improvement. Health posts readiness, ANC use, parity, and wealth status influenced the care-seeking behavior of mothers for their sick children.

## Introduction

Achieving adequate facility readiness to provide specific child health services especially at a population level is a complex process (1). But in concept it can be improved in terms of structural dimensions of quality; which is fulfilling important resources for the betterment of the process and outcome dimensions of quality (2). Facility readiness is an important first step to provide high quality services (3).

Through substantial investments in health facilities, Ethiopia has achieved prominent progress over the last decade in preventing child mortality and morbidity. Sick children benefit from high-impact interventions like integrated community case management (iCCM). However, neonatal mortality is still high (4). This is attributing to poor quality of services and low facility readiness. Lower level health facilities are positioned to improve access to quality child health services but the capacity and the readiness is often low (1).

Infection accounts for about half of all neonatal deaths in countries with the highest neonatal mortality rates. According to the World Health Organization (WHO), more than 2.5 million newborns died worldwide in 2017 due to infections, and there were 1.3–3.9 million cases of neonatal sepsis worldwide in 2018 (5). Infection was identified as a leading cause of mortality in sub-Saharan Africa, contributing to 37% of the 2.1 million neonatal deaths (6). Ethiopia ranks among the top 10 countries in neonatal mortality, with over 100,000 newborn deaths each year. The neonatal mortality rate (NMR) in Ethiopia was 30 deaths per 1,000 live births with no significant decline over the last decade (7, 8).

World Health Organization recommends a simplified regimen (injectable and oral antibiotics) for community-based management of newborns with possible serious bacterial infection (PSBI) when referral to a hospital is not possible (9), which in principle is an effective strategy to treat PSBIs at the community level that leads to high service coverage and a lower case fatality rate (10–14).

However, the coverage of sick newborn care is low due to multiple factors. Among them are a low supply of essential drugs, suboptimal supportive supervision, and poor referral links (15, 16). According to one study, inadequate care is attributed to 60% of disease-related deaths. Moreover, the use of health services has been more influenced by the quality of care (17).

According to the findings of the Lancet Commission for high-quality health, poor-quality service is the cause of 61% of neonatal fatalities (18); and 1.7 million newborn lives could be saved each year by investing in access to quality care for every newborn, everywhere (19).

Implementation of integrated community case management (iCCM) of common childhood illnesses improved the uptake of care for sick young infants (SYIs) aged 0–59 days by improving access and creating demand. However, evidence showed that iCCM programs that are implemented in different settings are usually of low quality and lack standardization in terms of the availability of important resources, including competent service providers (20, 21). In other words the iCCM programs, which have intensive training, supervision, fulfilling equipment, and supply support, provide better quality care (20, 21). Care-seeking for sick and young infants (SYI) from health posts increased significantly in the context of iCCM. However, many reasons exist for not seeking care at health facilities, including lower-level ones such as health posts. Considering the disease as not severe (56.2%), financial constraints (28.6%), being busy to visit health facilities (6.0%), the health facility being closed while visited (3.2%), transportation problems (2.7%), and underestimating the service quality at the nearby health facility (2.2%) are among the prominent reasons for the decline to get care at health facilities (22).

Health facility readiness measures quality in health facilities through interactions of different domains that could assist in visualizing the capacity of health facilities to provide appropriate care and services if different interventions such as iCCM are in place. Neonatal survival remains a primary concern for mothers, and facility readiness plays a critical role in influencing care-seeking behaviors, underpinning maternal confidence in the quality of services available. Consequently, readiness is a key determinant of maternal perceptions of care quality and functions as a pivotal enabler in the complex decision-making process regarding whether, when, and where to seek care. Enhancing facility readiness has the potential to substantially increase maternal health service utilization by fostering trust, alleviating concerns, and improving both perceived and actual service quality (23). A study in low- and middle-income countries indicates higher readiness of health facilities is associated with a higher quality of care for sick children; for every 10% point increase in readiness score, quality increased by about 1% (24). However, health facility readiness at the community level care across the available evidences was inadequate for the management of sick children (25). Not only low facility readiness reported but also, the influence of health facility readiness on mothers’ care-seeking practices for their sick children is not known, especially in the iCCM context; While many strategies including iCCM have been developed to enhance healthcare access for underserved populations at the community level, they often do so with limited emphasis on quality, particularly in terms of facility readiness. Therefore, in this study, we assessed the influence of health posts’ readiness on maternal care-seeking practices for their sick young infants in the context of iCCM in Ethiopia.

## Methods

### Context

Ethiopia is organized into 12 regional states and two autonomous city administrations, further divided into zones, districts (woredas), and villages (Kebeles which are the lowest administrative units). On the other hand, the healthcare system operates at three levels: primary, secondary, and tertiary. The primary level, consisting of health posts (HPs), health centers (HCs), and primary hospitals, provides community-based services close to residents. Each Kebele has at least one HP staffed by two female health extension workers (HEWs)—low-level healthcare workers with 10-month training mainly on disease prevention and cure to selected diseases. The secondary and tertiary levels focus on curative services and serve as referral centers. This study used data obtained from primary-level health facilities as part of implementation research under the JSI project, conducted between November 2020 and June 2022 in collaboration with woreda health offices and health centers in Lume and Dembecha werdas. The study aimed to improve the care-seeking behavior of mothers for their sick children and the community-level health services through the national iCCM strategy the details of which can be found in previously published works (26, 27). The iCCM is designed to enhance the care-seeking behavior of mothers for their sick children and to improve community-level health services; by linking women to the primary level health facilities. Key implementation strategies included on-site coaching and training for HEWs, facilitated referrals between health posts and health centers, strengthening supply chains, integrating PSBI treatment, and promoting early maternal and newborn care through pregnancy identification, home visits, delivery notifications, and postnatal care visits.

The study was conducted in two woredas (where the JSI project implementation site), in Ethiopia: Dembecha in the Amhara region and Lume in the Oromia region. Dembecha, located 349 km northwest of Addis Ababa, has a population of 129,260, with 13.9% living in urban areas. It includes 31 rural Kebeles, 31 HPs, and 6 HCs. Lume, situated 64 km east of Addis Ababa, has a population of 117,080, with 33.06% urban residents. It comprises 35 rural Kebeles, 35 HPs, and 7 HCs. For the study, 11 health centers and all 66 health posts across both districts were utilized (28),

### Data

This study analyzed data obtained from a population-based cross-sectional study involving before and after implementation interviews without a control arm representing the two woredas from two regions of Ethiopia; collected by JSI from 18 April-24 May 2021 for baseline, and from 06 June-09 July 2022 for the end line. The data collection includes household surveys and HP observation assessments. In the household surveys, interviews were done among mothers who had live births within the previous 2–14 months. We used the baseline and end-line data to see the changes in the numbers of sick children who needed treatment and sought care from their mothers for their sickness. The end-line data constituted facility-level and individual-level factors that affect the care-seeking practice of mothers for their sick young infants.

### Sampling and data collection

The sample size for all objectives of the project is calculated considering the following assumptions: 95% Confidence level (*Z*_α/2_ = 1.96), design effect (*D* = 1.5), and power of 80%, the proportion (p) 56% of sick children sought medical care (29), and the odds of care seeking among mothers who attended antenatal care (AOR = 1.68, 95% CI: 1.59–1.77); which gives the calculated sample size of 395 sick young infant cases that need care.

The survey used a two-stage stratified cluster sampling method, stratified by administrative regions and woredas. Interviews were conducted with all mothers whose children were under 15 months old. All households with children less than 15 months were recruited and eligible mothers were included in the study. Accordingly, 4,081 mothers with infants 2–14 months were identified and interviewed in the end line survey and 4,262 were in the baseline. Among all interviewed mothers during the end-line survey 359 mothers with sick young infants were identified and interviewed. For the health facility assessment, 11 HC and 64 HPs were included. Data were gathered through face-to-face interviews with mothers. During the interview, household and sociodemographic characteristics of mothers, awareness, and access to health services, and experiences of mothers related to the use of maternal health services were collected. The questionnaire used for the interview was translated into local languages (Amharic and Afan-Oromo).

### Measurements

To measure the care-seeking practice of mothers, interviews with mothers were about whether or not they sought care for their sick child outside of their home while the child was sick. To assess health posts’ readiness for the prevention and management of sick children, a review of the activity and observation checklists were adapted from the WHO Service Availability and Readiness Assessment (SARA) tool (30). The level of readiness was rated based on all questions asked and/or observed. The preparedness of facilities was measured in five domains including the availability of an adequate number of competent healthcare providers (i.e., healthcare providers who take trainings on iCCM and CBNC for management of sick children at the lower level health facilities, the availability of drugs, availability of equipment and supplies, availability of an effective referral system, and the availability of performance monitoring and support system were considered). One of the important approaches for implementing the iCCM strategy is to provide care or arrange referral to higher-level care as soon as possible during labor and PNC periods.

The level of facility readiness for the management of sick children was measured with the mean availability of tracer items across the five domains of readiness by assigning equal weight to each of the items. The overall service readiness score was calculated by computing the scores of the five domains; that provided the summated mean scores. Then to make our measurements comparable with other studies measured with different scales we calculated the maximum scale score. The wealth statuses of mothers were measured using the principal component analysis (PCA) and the other independent variables were measured by an interview of women for the presence or absence of each characteristic (Table 1).

**Table 1:** Description and measurement of variables, for the study, 2022.

Characteristics	Descriptions	Measurements
Complete ANC services utilization	It is defined as having at least 4 health facility visits during pregnancy for check-ups by skilled attendants	Categorized as having 4 plus ANC visits and less than 4 ANC visits
Skilled facility delivery (SFD)	It is defined as women who were assisted by a doctor, nurse, midwife, and HEW at a health facility like a hospital, HC, private hospital, clinic, or HP with gynecological and obstetric skills during their last pregnancy	Categorized as received SFD or not by identifying the place of delivery and interviewing women about the primary person who assisted their delivery
Postnatal care (PNC)	It is defined as women and their newborns who receive postpartum care at the health facility within 6 weeks of delivery	Measured by an interview of women if any health care professionals check their and their newborns’ health within 6 weeks of giving birth
Care seeking of mothers for their sick children	If mothers seek care for their sick children outside of their home due to any reason	It is measured through interviews with women to determine whether or not they seek care for their sick child while they are sick
Birth notification	It is a strategy introduced to promote early postnatal care	Measured through interviews of women if they inform the HEWs about their childbirth immediately after delivery
Pregnant women’s conference	It is a pregnant women’s meeting for peer learning to seek maternal and newborn health care, which is facilitated by health care providers	It is measured by asking women about their attendance at least one conference during their last pregnancy
The overall health posts readiness	Calculated from five readiness domains, availability of human resources, drugs and supplies, service provision materials, effective referral system, and performance monitoring and support system	The HP readiness score was calculated from the five domains and the summated mean score was calculated and converted into a maximum scale score
Severe bacterial infection	Difficulty breathing, chest in-drawing, not feeding well, unusually hot or cold, less active than usual, and/or convulsions	It is measured by asking women if any of the severity signs were experienced by their child during their last pregnancy
Household wealth index	A score constructed for each household from the household’s possessions with the principal component analysis	The household assets were computed by using PCA. And categorized into five groups
Walking distance to the nearest facility	Walking distance to the nearest health posts or health centers	Measured through the time taken to reach the nearest health posts or health centers

### Data analysis

To create a more complete dataset, we combined the facility-level data with the individual-level data using the unique identification given to each health facility. For this study, the mothers’ care-seeking practice for their sick children was taken as the dependent variable, and facility readiness is an independent variable measured using healthcare workers’ competence, drug availability, service provision material availability, the presence of an effective referral system, and the existence of performance monitoring and support system. Moreover, sociodemographic characteristics (maternal age, maternal education, paternal education, and marital status, sex of the child, gravidity, and parity), household wealth status, ANC follow-up, SBA/SFD use, and PNC use were considered confounding variables.

The wealth index of the household was determined using PCA. The level of health facility readiness was determined by calculating the proportions of the availability of tracer items for the five domains. The tracer items were coded as 1 if the item is available and 0 if not available. The item reliability was checked by calculating Chronbach’s alpha and the internal consistency of the tracer item for each domain was found to be in the acceptable range. We computed the readiness score by assigning equal weight for each of the tracer items for each domain. The overall readiness score was calculated from the five domains as a summated mean score. Then the summated mean score was standardized to the maximum scale score [i.e., converting summated scores (total of individual item scores)] into a standardized percentage to compare the results with others measured on different scales.


$$\text{Percentage Mean Score}=\frac{\begin{array}{l} \text{Summated Mean Score}-\\ \text{Score Minimum}\;\;\; \end{array}}{\begin{array}{l} \text{Score Maximum}-\\ \text{Score Minimum} \end{array}}*100$$

We did an independent sample *t*-test with a 95% CI and *p*-value < 0.05 to see the change in the care-seeking practice of mothers for their sick young infants during the implementation periods. To see the contributions of facility readiness and controlling confounding, we fitted a logistic regression. The crude odds ratios (COR) were calculated to select the candidate variables for multivariable analysis. Variables with a *p* < 0.25 for the COR were considered as candidates for multivariable regression analysis. In multivariable analysis, maximum likelihood estimation techniques were used. For the final model, multivariable logistic regression analysis with log link estimation techniques was done to identify the independent predictors of the care-seeking practice of mothers for their SYIs and to see the contributions of facility readiness for the care-seeking practice of mothers for their SYIs. The respective AOR at *p* < 0.05 and 95% confidence intervals were used to declare variables that have a statistically significant association.

### Ethical approval

Ethical clearance was obtained from the Ethiopian Public Health Association (EPHA) Research Ethics Review Committee (Reference #: EPHA/OG/166/21 dated April 16, 2021) and renewed for the end-line survey (Reference #: EPHA/OG/781/22 dated April 18, 2022). Informed consent was obtained from all participants after providing them with comprehensive information about the research to ensure they fully understood the study. The consent form was approved by the IRB, and voluntary participation was ensured throughout the study. All participants were informed about the study’s purpose, benefits, and potential risks, and they were made aware of their right to opt out to answer any questions. When a respondent agreed to participate after reviewing the consent information, the interviewer documented consent by marking the questionnaire and providing a digital signature below the consent statement. Interviews proceeded only after consent was given and properly documented. For the facility assessment permission letters were obtained from the Woreda health offices. The researchers ensured that all information obtained from participants was kept confidential and private to respect their privacy and maintain the integrity of the study. All respondent identifiers were kept confidential, and data were anonymous. The authors confirm that all methods were performed in accordance with the relevant guidelines and regulations such as the Declaration of Helsinki and the Ethiopian National Research Ethics Review Guideline.

## Results

### Socio-demographic characteristics

In the household survey, More than two-thirds of the respondents were 20–34 years old. About 1,518 (70%), had primary education, about 2,788 (68.3%) of them lived less than 30 min walking distance to the nearest health post, and 3,750 (91.9%) of them lived less than 2 h walking distance to the nearest health center Among the 64 HPs, 85.9% of them have trained HEWs, 59.4% of HPs have two or more HEWs, and 40.6% of them have only one HEW during the facility visit (Table 2).

**Table 2:** Socio-demographic characteristics of the study population, 2022.

Characteristics		*N* = 4,081
District	Denbecha	2,634 (64.5)
	Lume	1,447 (35.5)
Maternal age in completed years during the birth of this child	<20	167 (4.1)
	20–34	2,818 (69.1)
	35–49	1,096 (26.9)
The highest grade the mother completed	No education	2,218 (54.3)
	Primary	1,239 (66.5)
	Secondary	535 (28.7)
	Higher	89 (4.8)
Maternal religion	Orthodox	3,884 (95.2)
	Protestant	156 (3.8)
	Muslim	26 (0.6)
	Waaqeffanna	15 (0.4)
Marital status	Married	3,955 (96.9)
	Never married	17 (0.4)
	Widowed	13 (0.3)
	Divorced/separated	96 (2.4)
The highest grade the husband completed	No education	1,913 (46.9)
	Primary	1,518 (70)
	Secondary	522 (24.1)
	Higher	128 (5.9)
Sex of this child	Male	2,129 (52.3)
	Female	1,944 (47.7)
Have a live birth before this infant	Yes	2,960 (72.7)
	No	1,112 (27.3)
Number of pregnancy (gravidity)	Primigravida	43 (1.1)
	Multigravida	1,963 (48.1)
	Grand multigravida	1,077 (26.4)
	Great grand multigravida	998 (24.5)
The birth order of this child	1st	1,061 (26.0)
	2nd	898 (22.0)
	3rd and above	2,122 (52.0)
Wealth status	Poorest	853 (20.9)
	Poorer	777 (19.0)
	Medium	855 (21.0)
	Richer	768 (18.8)
	Richest	828 (20.3)
Distance to the nearest health post	≤30 min	2,788 (68.3)
	>30 min	1,293 (31.7)
Distance to the nearest health center	≤2 h	3,750 (91.9)
	>2 h	331 (8.1)

### Facility readiness

#### Availability of adequate numbers and competent healthcare providers

The mean readiness score for the availability of adequate numbers and competent healthcare providers was 74.6%(95%CI: 73.8–80.4). About 40.6% of HPs have less than the mean healthcare providers readiness score, and about 59.4% of HPs have more than the mean readiness score. In this regard, all HPs have HEW, 59.4% of HPs had more than one HEW who was working in each HP, and 40.6% of HPs had only one HEW. About 85.9% of HPs had at least one trained HEW (i.e., HEWs who gets special trainings about iCCM) for the management of sick children at the lower level health facilities (Table 3).

**Table 3:** Health posts readiness domains and tracer items for management of sick children in Denbecha and Lume districts, Ethiopia, June 2022.

Readiness domains	Tracer items	Percentages
Human resource	Adequate number of HEW are available in each HP	59.4
	Had trained and/or level 4 HE	85.9
	Had enough or close mentorship	43.8
	Had level 4 HEW	85.9
	HEW able to provide iCCM/CBNC services	98.4
	**Mean readiness score for tracer items**	**74.6**
Drugs and medical supplies availability	Paracetamol	31.3
	2 cc syringe and needle	59.4
	Gentamicin 20 mg injection	70.3
	Chloroquine	71.9
	Rapid diagnostic test (RDT)	79.7
	RUTF (Plumpy Nut or BP100) Sachets	79.7
	Zinc	81.3
	Face mask	81.3
	Examination gloves	82.8
	Coartem (artemether-lumefantrine)	85.9
	Alcohol hand rub or sanitizer	87.5
	ORS	89.1
	Albendazole/Mebendazole	96.9
	Amoxicillin dispersible tablet	98.4
	Vitamin A	98.4
	**Mean readiness score for tracer items**	**79.3**
Availability of service provisions materials	Ambu-bag and masks	12.5
	Suction	21.9
	Newborn registration book	54.7
	Functional weighing scale	67.2
	Pregnancy registration book	73.4
	Functional thermometer	93.8
	MUAC tape	98.4
	**Mean readiness score for materials availability tracer items**	**60.3**
Effective referral system	The community have direct call system to access an ambulance	43.8
	Standard protocols available for sick infants referral	53.1
	The HPs have standardized referral slips for sick newborn referral	53.1
	Have access to an ambulance or vehicle for newborn emergencies	62.5
	Have standardized referral-out registers for sick newborn	65.6
	HEWs have cellphone for communication	93.8
	Communities or WDAs have HEWs’ phone for emergency call	92.2
	**Mean readiness score for effective referral tracer items**	**62.3**
Performance monitoring and Support system readiness	This facility received a supervision visit from the higher level (WorHO, ZHD, RHB, MOH or partners)	21.9
	Received support from the PHCU’s workers like midwife, nurse or health officers	85.9
	Frequent support received from PHCU’s workers	78.0
	Had meetings with different stakeholders to get support	65.6
	participate in a PHCU level performance review and clinical mentoring meeting meet in the last 6 months	62.5
	Have any form of QI interventions	32.8
	Have functional QI team (meets regularly to review quality of activities and projects)	23.4
	Have facilitated family conversations in the last 6 months	50
	There is a pregnancy notification system in the Kebele for early ANC booking	84.4
	Used any reminders for pregnant women to come for their subsequent ANC visits	85.9
	Have birth notification system	84
	Conduct women’s conference regularly	96.9
	Conducted any orientation meetings with the Kebele managers to prioritize iCCM	67.2
	Working with Kebele managers to make WDA structure functional	82.8
	Facilitate Kebele-level multi-sectorial meetings to get support	62.5
	**Mean readiness score for Performance monitoring and Support system readiness tracer items**	**65.3**

The bold text indicates the mean readiness score for each domain, computed from the tracer items under each readiness domain.

### Drugs and medical supplies availability

Health posts readiness for the management of sick children in terms of the availability of drugs and medical supplies was 79.3% (95%CI: 78.7–83.2%). About 28 (43.8%) of HPs have less than the mean readiness score, and 36 (56.3%) of HPs have a readiness score greater than the mean. Amoxicillin was available in 63 (98.4%) of HPs, Gentamicin (20 mg injection) was available in 45 (70.3%) of HPs, and Albendazole or Mebendazole was available in 62 (96.9%) of HPs. Whereas Paracetamol was available in only 31.3% of HPs (Table 3).

### Availability of materials/equipment to provide healthcare service

Health posts readiness for the management of sick children in terms of the availability of materials used to provide healthcare was 60.3% (95%CI: 55–65.4%). Most health posts (98.4%) have functional thermometry, and the middle Upper Arm Circumference (MUAC) measurement apparatus is available in 93.8% of health posts. All HPs have an iCCM and/or Community-Based Newborn Care (CBNC) Chart booklet; 76.6% of HPs have functional blood pressure measuring apparatus. Whereas only 21.9 Health posts have suction and only 12.5% of HPs have Ambu bags and masks (Table 3).

### Effective referral

Health posts’ readiness for the management of sick children in terms of the availability of an effective referral system was 62.3% (95%CI: 56.7–68.1). About 39.1% of HPs have a lower mean readiness score, and 60.9% of HPs have a greater mean readiness score. In 93.8% of HPs HEWs have cellphones and 92.2% of them give their phone numbers to the women development arms (WDAs), or the pregnant women for emergency calls. About 62.5% of HPs have access to an ambulance or another vehicle for newborns, and 65.6% of HPs have a functioning cellular telephone or a private cellular phone that is supported by the HC/district health office to facilitate the referral. About half of 53.1% of HPs have standard protocols for sick newborns’ referral (for whom, when, and where to refer) (Table 3).

### Performance monitoring and support system readiness

Health posts readiness for the management of sick children with regards to performance monitoring and support system was 65.3% (95%CI: 60.3–71.3). About 85.9% of HPs had received integrated supervision from midwives, nurses, or health officers including the iCCM program for the management of SYIs. However, only 21.9% of HPs received supervisory visits from higher-level officials (district health office, zonal health department, regional health bureau, ministry of health, or partners). In 96.9% of HPs, HEWs conduct women’s conferences regularly, and in 84.4% of HPs, there was a pregnancy notification system. Whereas only 32.8% of HPs have quality improvement interventions and 67.2% of HPs conducted orientation meetings with the Kebele managers to prioritize iCCM. However, only 32.8% of HPs have any form of quality improvement (QI) interventions, and 23.4% have functional QI teams (Table 3).

### Overall health facility readiness

**Figure 1:**
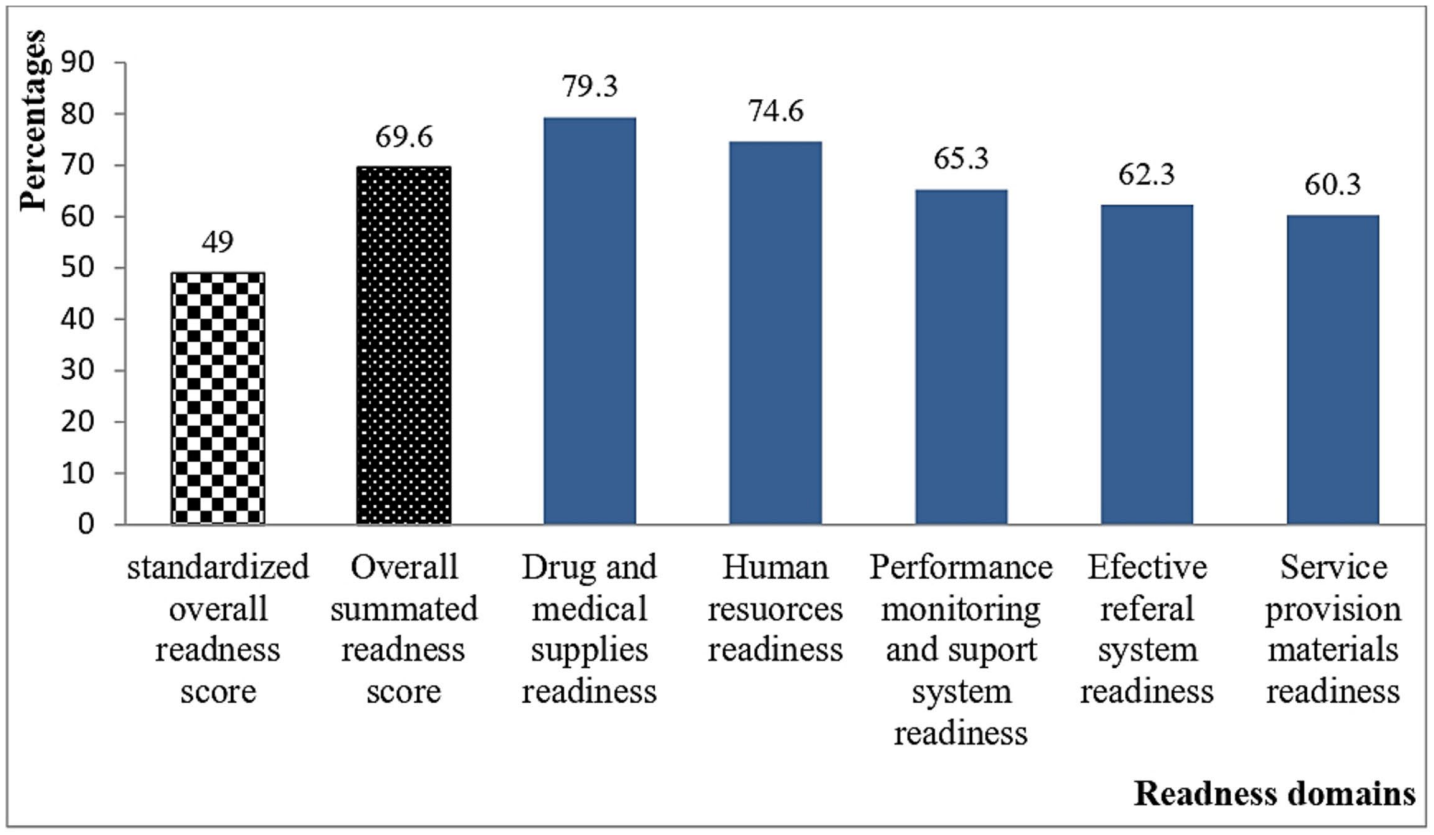
The overall health posts readiness score measured in six domains in Denbecha and Lume districts, Ethiopia: June, 2022.

The highest readiness score were 79.3%, the lowest readiness score were 60.3% and the overall summated mean score was 69.6% (95%CI: 66.2–72.8). However, as it is standardized using the maximum scale score it was only 49.0%. Among the five domains, the availability of drugs and medical supplies has the relatively highest readiness score (79.3%). Whereas, effective referral system and availability of service provisions materials domains had the lowest scores with 60.3 and 62.3%, respectively (Figure 1).

### Maternal health service utilization

Most mothers 3,471 (85.5%) had ANC follow-up visits at a health facility. Among them, nearly half, 1,657 (47.7%) of mothers initiated early ANC within 12 weeks of pregnancy, and 1,744 (50.2%) had ANC4 + visits. Two-thirds of mothers, 2,726 (66.8%), gave birth in health facilities with a skilled provider. About 1,795 (44.0%) of them were assisted by midwives and nurses, 387 (9.5%) by doctors, and 24 (0.59%) by HEWs. However, a significant number, 1,175 (28.8%), of deliveries were assisted by nonprofessional relatives and friends. About 1,318 (32.4%) of mothers had at least one PNC visit; 1,120 (27.5%) did PNC visits for their third child and 995 (24.4%) had PNC for themselves. About 650 (15.9%) of mothers read or discussed about family health card (FHC) and only 298 (7.3%) of mothers had FHC. One-third, 1,367 (33.5%), of mothers attended pregnant women’s conferences (Table 4).

**Table 4:** Maternal health service utilization in Denbecha and Lume districts, Ethiopia, June 2022.

Characteristics		*n* = 4,081 (%)
Use ANC for this pregnancy	Yes	3,471 (85.9)
	No	568 (13.9)
	Not sure	42 (1.0)
Early ANC booking (within 12 weeks of pregnancy)	Yes	1,657 (40.6)
	No	1,814 (44.5)
Numbers of ANC visit for this pregnancy	Less than 4 ANC visits	1,727 (42.3)
	ANC 4 + visits	1,744 (42.7)
The place where received ANC for this pregnancy	Hospital	300 (8.6)
	Health Center	3,299 (95.0)
	HP	817 (23.5)
	NGO health facility	66 (1.9)
	Private facility	239 (6.8)
	Total	3,471 (100)
Received pregnancy care from a health post	Yes	1,162 (28.5)
	No	2,919 (71.5)
Community health workers visit you during pregnancy	Yes	812 (19.9)
	No	3,167 (77.6)
	I do not remember	102 (2.5)
Ever attend pregnant women’s’ conference	Yes	1,367 (33.5)
	No	2,714 (66.5)
Have birth preparedness	Yes	3,283 (80.4)
	No	798 (19.6)
Mothers give birth at health facility with skilled provider	Yes	2,726 (66.8)
	No	1,355 (33.2)
The primary person that assisted your delivery	Doctor	387 (9.5)
	Nurse/midwife	1,795 (44.0)
	HEW	24 (0.6)
	TBA	118 (2.9)
	Relative/friend	1,175 (28.8)
	Nobody	22 (0.5)
Use PNC in the post-partum period	Yes	1,318 (32.4)
	No	2,763 (67.6)
PNC checkups were take place at	Own home	205 (18.3%)
	Health post	215 (19.2%)
	Hospital	185 (16.5%)
	Health center	432 (38.6%).

### Changes in mothers’ care-seeking practice at health posts for their sick children

**Figure 2:**
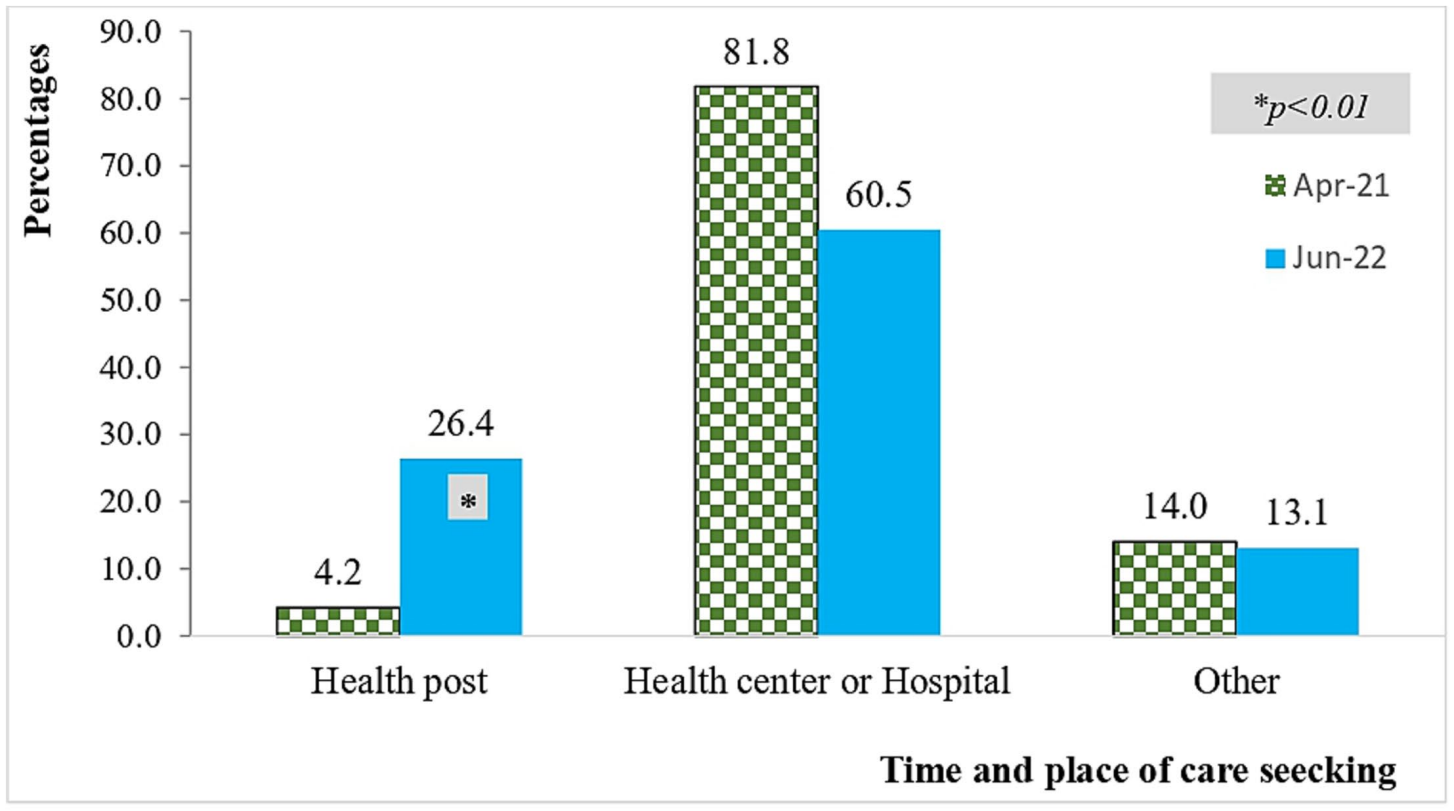
Change in distribution of place of care seeking and time of care for sick children.

Approximately 26.4% of mothers sought care for severe infections at health posts (HPs) during the end-line study, representing a significant increase from the baseline figure of 4.2%. About 459 children were sick of whom 359 (78.2%) were young infants in the end-line survey. About 316 (88.0%) mothers with sick infants sought care in the end line survey compared with only 291 (57.3%) who did it in the base line survey. About 45.6% of SYI cases received care at health centers and 23.4% received care at health posts in the end line survey where as 69.8% of SYI cases received care at the health centers and 4.5% received care at health posts in the baseline survey indicating a significantly higher change in care seeking at health posts in the end line survey (Figure 2).

Data from the facility records also showed that, many sick children visited HPs and received appropriate treatment at the health post level. About 955 pneumonia cases were correctly evaluated in the last 8 months (from November 2021 to May 2022). From diagnosed children with pneumonia, about 912 (91.6%) of sick children were properly treated (i.e., took the correct dose of medication, adhered to the schedule, and followed the recommended duration of treatment) among cases seen in the last 8 months. Moreover, from 45 very severe diseases (VSD), 39 (86.7%) were correctly treated (i.e., take the correct dose of medication, adhere to the schedule, and follow the recommended duration of treatment) within the last 8 months from November 2021 to May 2022 (Figure 2).

### The correlation between health posts readiness and care-seeking practice of mothers for their sick children

In bi-variable analysis, maternal age during the birth of the index child, district type, maternal education, religion, wealth status, parity, walking distance to the nearest health post, distance to the nearest health center, ANC use, PNC use, pregnant women’s conference attendance, health facility readiness in terms of human resources, drug availability, service provision material availability, support system readiness, performance monitoring readiness, and effective referral availability readiness were associated with the healthcare seeking of mothers for their sick children, at a *p*-value of < 0.25. On the other hand, wealth status, district type, parity, distance to the nearest HP, ANC use, health facility readiness in terms of service provision material availability, effective referral system availably, performance monitoring system readiness, and support system readiness had a statistically significant association with the healthcare seeking practice of mother for their young infants in multivariable analysis at a *p*-value of < 0.05 and 95% CI. The odds of healthcare seeking among mothers for their sick children in Lume district were 87% times lower than that of Denbecha district mothers to seek healthcare for children (AOR = 0.13: 95%CI; 0.1–0.3), and the odds of healthcare seeking among grand multipara mothers were 73% lower than primi-para women (AOR = 0.27: 95%CI; 0.1–0.7). The odds of care seeking for sick children among mothers who are members of rich households were 2.02 times higher compared to those living in poor households (AOR = 2.02: 95%CI; 1.1–3.9). The odds of care seeking for sick children among mothers who used ANC were 2.76 times higher compared to those who did not have ANC visits (AOR = 2.76: 95%CI; 1.3–5.7). The odds of healthcare seeking for sick children among mothers who had PNC visits were 41% lower than those who did not have PNC visits (AOR = 0.59 95%CI: 0.36–0.96). For every one-unit increase in the readiness score with the availability of materials and medical supplies, the odds of health-seeking care among mothers increased by 52% (AOR = 1.52: 95%CI; 1.2–1.9). Whereas, for every one-unit increase in the readiness score with the availability of an effective referral system, the odds of healthcare-seeking for sick children decreased by 19% (AOR = 0.81: 95%CI; 0.7–0.97). And for every one-unit increase in human resource readiness score the care-seeking practice of mothers decreased by 28% (AOR = 0.72 95%CI: 0.54–0.96) (Table 5).

**Table 5:** Factors associated with the healthcare-seeking practice of mothers for their sick children in Denbecha and Lume districts Ethiopia 2022.

Characteristic		Care seeking for sick young infants			
		Yes	No	COR (95%CI)	AOR (95%CI)
District	Denbecha	252	72	1	**1**
	Lume	64	72	0.26 (0.2–0.4)	0.13 (0.1–0.3)**
Maternal age during the birth of this child				0.97 (0.9–1.01)	0.98 (0.9–1.04)
Religion	Orthodox	306	131	2.8 (1.18–6.65)	1.31 (0.47–3.63)
	Others	10	12		1
Maternal education	Primary	114	44	1	1
	Secondary	46	23	0.77 (0.4–1.4)	1.05 (0.5–2.3)
	Higher	6	8	0.3 (0.1–0.9)	0.39 (0.1–1.7)
Parity	Primi-para	104	34	1	1
	Multipara	153	72	0.7 (0.4–1.1)	0.60 (0.3–1.2)
	Grand multipara	58	35	0.5 (0.31–0.96)	0.27 (0.1–0.7)*
	Great grand multipara	1	2	0.2 (0.01–1.9)	0.14 (0.01–2.1)
Wealth status	Poor	150	63	1	1
	Medium	67	21	1.3 (0.8–2.4)	1.8 (0.9–3.7)
	Rich	99	59	0.7 (0.44–1.09)	2.02 (1.1–3.9)*
Distance to the nearest HP	<30 min	199	115	1	1
	>30 min	117	28	2.4 (1.5–3.9)	2.43 (1.4–4.3)*
ANC use	Yes	272	119	1.5 (0.87–2.7)	2.35 (1.2–4.7)*
	No	36	24	1	1
PNC use	Yes	98	67	0.51 (0.3–0.8)	0.59 (0.36–0.96)*
	No	218	76	1	1
Attended pregnant women conference	Yes	102	43	1.11 (0.72–1.7)	1.58 (0.91–2.74)
	No	102 |	100	1	1
Human resource readiness		0.82 (0.66–1.01)			0.72 (0.54–0.96)*
Materials availability readiness		1.16 (1.02–1.31)			1.52 (1.2–1.9)**
Effective referral availability readiness		0.81 (0.71–0.93)			0.81 (0.7–0.97)*
Performance monitoring and support system readiness		0.92 (0.85–0 0.99)			0.93 (0.9–1.0)

The bold text indicates variables that have a statistically significant association with the outcome variable. Specifically, ** denotes a statistically significant association at *p* < 0.001, * statistically significant association at *p* < 0.05.

## Discussion

This study identified the suboptimal readiness of health posts in Ethiopia to provide adequate care for sick children. Mothers’ care-seeking behavior for SYIs was significantly influenced by factors such as the material readiness of health facilities, ANC use, parity, wealth status, administrative woreda, and walking distance to health facilities. In contrast, PNC use, human resource readiness, and the availability of effective referral systems were found to be negatively associated with care-seeking behavior. The readiness of health posts influence mothers’ care-seeking practices for their sick children. The findings of this study highlight a significant improvement in mothers’ care-seeking practices, increasing from 57.3% at baseline to 88.0% at the end-line survey (*p* < 0.001), reflecting changes over the implementation period. These findings are higher than those of a previous study in Bangladesh, where most mothers only brought their children to the hospital lately after the child developed danger signs, severe forms of the disease, or associated complications (31). This might be attributed to the readiness of health posts, improved availability of drugs and medical supplies, and improved mothers’ care-seeking practices at the community level in Ethiopia. Health facility readiness influences mothers’ care-seeking behavior to offer essential child health treatments is a complex process. Essential child health care requires skilled healthcare professionals who can provide the necessary services in an environment with adequate infrastructure, medications, supplies, and equipment. Facility readiness on its own is insufficient for providing high-quality services (3); however, it is an important first step (1).

Subsequently, in this study, we found suboptimal level of overall readiness score, indicating an important snapshot of how prepared healthcare facilities are to manage sick children. Our study shows lower readiness scores compared to previous studies conducted in India (1). It is also lower than a study done in Timor, the readiness score increased from 44 to 89% after the implementation of health improvement projects at health posts, health centers, and hospitals (32). The gap may be due to differences in socio-economic status about healthcare infrastructure and resource availability. And, the implementation strategies in our context more focus on the accessibility of important drugs than on improving the overall quality dimensions. Our study is similar to studies conducted in Nepal, where the mean readiness score was 52.1%, and only 50% of facilities were ready to offer sick child care (33). It may be because in Nepal, many sick children have access to hospital care, and they ignore the fulfillment of supplies at the community level.

In this study, we identified a moderate level (74.6%) of HP readiness about the availability of adequate numbers and competent healthcare providers. This is better than a study done in Kenya that demonstrated providers at PHC units, lacked effective specialized knowledge and/or basic skills for the management of sick children (34). However, it is lower than the study done in Bangladesh, where all service providers received at least a 5-day training session on the infection management guidelines, and 63–97.7% of providers were able to classify PSBI (35). In our study, about 40.6% of HPs have only one HEW, and only 59.4% of HPs have more than one HEW in each HP. This is against the Ethiopian Health Extension Program (HEP); the implementation guidelines stipulate a staffing pattern of two HEWs for each HP (36).

Our study also identified a moderate level of 79.3% of HP readiness concerning the availability of drugs and supplies. Which is higher than the study done in Malawi (37) and India where essential drugs and supplies readiness scores increased from 52to 73% after the implementation of the intervention (1). Even though our study focused on primary health care units at the community level, it had the same result with Malawi that included all health facilities from the lower to the referral levels. This may be due to the implementation strategies that improved the facility readiness at the community level better than the hospital level readiness. This study is also higher than a nationwide service availability and readiness assessment done in Ethiopia (38). This may be due to the interventions in our study had improved the facility’s readiness (27).

A relatively lower readiness score was observed with the availability of service provision materials 60.3% and effective referral system 62.3% compared to all HP readiness domains. The readiness of HPs to successfully refer clients to the right services determines the life of the child. Delays in referral determine the life and death of the newborn because time is crucial for them. Poor referral systems due to the scarcity of resources, including healthcare providers, and a lack of communication technologies result in ineffective referral communications between facilities (39). Moreover, the receiving facility is expected to anticipate the arrival, provide care and follow-up for the patient, and send back the referral form and feedback to the initiating facility to confirm or disprove the appropriateness of the referral (39). This is similar to another nationwide service availability and readiness assessment done in Ethiopia, which shows the mean availability of tracer items for service provision materials was 63% and even lower at the health post level, which is 57% (38).

The performance monitoring and support system, a readiness score of 65.3% in this study indicates moderate readiness, suggesting that while some elements of a sound performance monitoring and support system are in place, there is room for improvement. This may involve the need for more consistent monitoring and support or enhanced training for staff involved in performance monitoring and supportive supervision. In more developed healthcare systems, performance monitoring systems generally have higher readiness scores and contribute to improved health outcomes within the context of shared responsibility and accountability for achieving desired results (40). The gap in this regard may be due to limited access to technology, insufficient training, and a lack of standardized monitoring practices are there.

This study analyzed the factors affecting mothers’ care-seeking practices for their sick children outside the home. The results indicate that after controlling for confounding variables, wealth status, maternal parity, district type, distance to the nearest healthcare facility, antenatal care utilization, healthcare provider readiness with available materials and medical supplies, an effective referral system, a strong support system, and a performance monitoring system are predictors of mothers’ care-seeking practices for their children.

Our analysis indicates a significant positive association between the health posts readiness and care-seeking practice of mothers for their sick children after controlling confounding variables. For every one-unit increase in the readiness score with availability of materials and medical supplies, the odds of seeking care among mothers increase by 52 %. This is similar to other studies done in Malawi: that highlight better facility quality is positively associated with the utilization of sick child healthcare services (37). This suggests improving the availability of medical supplies and materials in health facilities could substantially enhance the likelihood of care-seeking. When health facilities are well-prepared with essential materials and medical supplies, mothers are more likely to seek care for their children. This study remarks that an increase in the readiness score of HPs reflects how well-prepared they are to provide appropriate care, and increases the likelihood of mothers seeking care.

Whereas, against previous studies (15, 41); facility readiness with human resources and referral system were negatively associated with care care-seeking practice of mothers outside their homes. This may be due to mothers feeling more confident in the referral system, ensuring quick access to support and the appropriate level of care if needed. However, the particular intervention reduces the need to seek healthcare outside their home for every sickness. It also minimizes the need for mothers to travel long distances for care, as they can trust the local support system and monitoring system. Another reason could be cultural and social considerations. In some contexts, strong community support systems align with cultural preferences for seeking care within the community. Mothers may prefer staying at home due to these cultural and social factors (15). Another possible explanation is that unlike other studies the data collection period, which occurred between 2021 and 2022, coincided with the peak of the COVID-19 pandemic. During this time, mothers were advised to stay at home unless they faced serious medical conditions.

The odds of care seeking among women who were from rich wealth status were 2.02 times higher compared with poor families. This is in line with a study done in Nigeria (42). However, it contrasts to the findings of a study done in southern Ethiopia (43). The disagreement might be explained by the fact that our study encourages every woman to seek care at all health facilities; whereas the previous study aimed to serve the poorest households at the health post and household levels to increase service access to richer families who can access health services at higher level health facilities. In our study, ANC utilization was associated with the care-seeking practice of mothers for their children. The result is consistent with a previous study done in northwest Ethiopia (44). This highlights that ANC visits provide opportunities for mothers to meet healthcare providers, and receive important information from healthcare providers about newborn danger signs and the need for child health care. In this study, grand multipara women were 73% less likely to seek care for their sick child compared to primi-para women. This might be explained by the fact that multi-Para women have experience and can identify critical illnesses that require treatment: whereas first-time mothers are more likely to be younger, which may make them more cautious and inclined to seek medical help (45).

## Limitations of the study

Despite its strength, this study has a limitation that the readers should consider; the basic amenities under the general service readiness items were not included in the health posts readiness assessment. And the study could have been more informative if it had been supplemented by a qualitative study.

## Conclusion

In this study a suboptimal level of HP preparedness highlights the need for continued efforts to improve service provision materials availability, effective referral system, performance monitoring and support system, and continuous tanning and development of health workers for the management of sick children at the community level, particularly in under-resourced areas. Health post readiness significantly influences the healthcare-seeking practice of mothers for their sick children. The readiness of HPs, particularly in terms of materials and medical supplies, is a major factor in determining the care-seeking behavior of mothers for their sick children outside their homes. Moreover, ANC use, parity and wealth status, and distance to the health facility are significantly associated with the healthcare-seeking practice of mothers for their sick children.

## Data Availability

The datasets used in this study are included in the manuscript and can be obtained from the principal investigator upon reasonable request.
